# Will the USA be able to endure another Trump presidency?

**DOI:** 10.1016/j.eclinm.2024.102613

**Published:** 2024-04-18

**Authors:** eClinicalMedicine

Trump became the 45th president of the United States of America after his inauguration in January 2017 and his term lasted until Jan 20, 2021, when President Biden took over office. On March 12, 2024, in his rerun for a second term, Trump won the Republican presidential nomination. The USA is now facing an election in November 2024, with 7 months of campaigning left for the candidates and the public. On Nov 5, 2024, voters will have to decide who they entrust to lead the nation for the next 4 years. Such trust should reflect the belief that the President will act in the best interest for the nation and the wellbeing of its people.

Looking back on the Trump presidency, many of his actions, policies, and executive orders were detrimental to the health and wellbeing of so many living in the USA. For decades now, the USA is lagging behind in the life expectancy compared with other high-income countries, such as Canada and the UK. US life expectancy was 2.2 years shorter than the average of other G7 countries, and, by 2018, the gap had widened to 3.4 years. Another way to look at this is to quantify the excess deaths, which is also called the number of missing Americans, representing the amount of avoidable deaths. By this mean, in 2018, the USA counted 461,000 avertable deaths, mostly in the younger than 65 years population.

President Trump’s negative effect on the health of Americans was exacerbated by his response to the COVID-19 pandemic. As many documented, President Trump’s public denial of the threat, dismissing international cooperation, and refusal to put a national response plan in action resulted in 450,000 deaths, as of early February 2021. 40% of these deaths could have been averted compared with the weighted average of the other G7 nations. President Trump’s personal failure caused substantial delays in accounting for equipment shortages and diagnostic testing. He politicised basic public health measures, such as wearing a face mask, physical distancing, and school closures, which contributed even further to the death toll due to COVID-19 in the USA. Asides from his reckless response to the COVID-19 pandemic, on Trump’s first day in office, he issued his first executive order on health-care financing with the intention to repeal the 2010 Affordable Care Act. Over the next years, President Trump’s actions and the reduction in Medicaid resulted in an additional 2.3 million uninsured US residents, including over 700,000 children.

Alongside the policies and executive orders that directly affected the health system and induced an immediate negative effect on the wellbeing of Americans, President Trump also enacted a number of policies with long-term effects, such as policies affecting food and nutrition, and the environment and global climate. Food safety and security were negatively affected during the Trump presidency. President Trump put several actions in place that loose standards for organic food production, blocked the endorsement for soda taxes, and weakened the largest anti-hunger programme (Supplemental Nutrition Assistance Program) in the USA. Furthermore, the Trump administration rolled back school meal standards allowing schools more flexibility on serving milk, whole grains, and the level of sodium options, which ultimately led to the US Department of Agriculture to announce weakened nutrition standards in early 2020. Regarding environmental and occupational regulations, Trump rolled back at least 84 protections laws, encouraged fossil fuel combustion and fracking, and withdrew from the Paris Climate Agreement. These changes were estimated to cause an additional 80,000 deaths with over 1 million people estimated to have exacerbated respiratory problems over the next decades.

As it is in US politics, and maybe luckily so, the Government, and thus the direction of the country, can swiftly change after a Presidential election due to the change of power. One could also argue that the democratic system was strong enough to voice a need for change from its citizens and with that President Biden took office on Jan 20, 2021. During his first 100 days in office President Biden reversed 42 of former President Trump’s policies mostly related to health and social justice, including stopping the withdraw from WHO, rejoining the Paris Agreement, expanding the eligibility for food assistance, and reopening the enrolment in health coverage through the Affordable Care Act.

In April 2024, a second Trump presidency is looming. We are already hearing similar rhetoric from the candidate Trump, as he continues his assault on medicine and science. Nothing has actually changed in his campaign; the banter and plain lies are still the same. His actions as President should have disqualified him to run again for office, not even considering his current legal issues and with that his potential conflict of interests. As it is put in the USA, it is “America’s choice”, and it will be. On Nov 5, 2024, we encourage US voters to scrutinise the track records of all candidates and vote for the individual who will work to ensure the health, wellbeing, and safety of all Americans. And Trump has proven, this is not what he has in mind.

